# Efficacy of Physiotherapy Interventions on Weaning in Mechanically Ventilated Critically Ill Patients: A Systematic Review and Meta-Analysis

**DOI:** 10.3389/fmed.2022.889218

**Published:** 2022-05-09

**Authors:** Lorenzo Lippi, Alessandro de Sire, Francesco D’Abrosca, Biagio Polla, Nicola Marotta, Luigi Mario Castello, Antonio Ammendolia, Claudio Molinari, Marco Invernizzi

**Affiliations:** ^1^Physical and Rehabilitative Medicine, Department of Health Sciences, University of Eastern Piedmont “A. Avogadro”, Novara, Italy; ^2^Translational Medicine, Dipartimento Attività Integrate Ricerca e Innovazione (DAIRI), Azienda Ospedaliera SS. Antonio e Biagio e Cesare Arrigo, Alessandria, Italy; ^3^Physical Medicine and Rehabilitation Unit, Department of Medical and Surgical Sciences, University of Catanzaro Magna Graecia, Catanzaro, Italy; ^4^Cardiopulmonary Rehabilitation Unit, Azienda Ospedaliera SS. Antonio e Biagio e Cesare Arrigo, Alessandria, Italy; ^5^Department of Translational Medicine, University of Eastern Piedmont “A. Avogadro”, Novara, Italy; ^6^Unit of Internal Medicine, Azienda Ospedaliera SS. Antonio e Biagio e Cesare Arrigo, Alessandria, Italy; ^7^Laboratory of Physiology, Department for Sustainable Development and Ecological Transition, University of Eastern Piedmont “A. Avogadro”, Novara, Italy

**Keywords:** mechanical ventilation, rehabilitation, weaning, intensive care, physiotherapy

## Abstract

Mechanical ventilation (MV) is currently considered a life-saving intervention. However, growing evidence highlighted that prolonged MV significantly affects functional outcomes and length of stay. In this scenario, controversies are still open about the optimal rehabilitation strategies for improving MV duration in ICU patients. In addition, the efficacy of physiotherapy interventions in critical ill patients without positive history of chronic respiratory conditions is still debated. Therefore, this systematic review of randomized controlled trials (RCTs) with meta-analysis aimed at characterizing the efficacy of a comprehensive physiotherapy intervention in critically ill patients. PubMed, Scopus, and Web of Science databases were systematically searched up to October 22, 2021 to identify RCTs assessing acute patients mechanical ventilated in ICU setting undergoing a rehabilitative intervention. The primary outcomes were MV duration, extubation, and weaning time. The secondary outcomes were weaning successful rate, respiratory function, ICU discharge rate and length of stay. Out of 2503 records, 12 studies were included in the present work. The meta-analysis performed in 6 RCTs showed a significant improvement in terms of MV duration (overall effect size: −3.23 days; 95% CI = −5.79, −0.67, *p* = 0.01; *Z* = 2.47) in patients treated with a comprehensive physiotherapy intervention including early mobilization, positioning, airway clearance techniques, lung expansion and respiratory muscle training. The quality assessment underlined 9 studies (75%) of good quality and 3 studies of fair quality according to the PEDro scale. In conclusion, our results provided previously unavailable data about the role of comprehensive physiotherapy intervention in improving MV duration in critical ill patients without chronic respiratory conditions. Further studies are needed to better characterize the optimal combination of rehabilitation strategies enhancing the improvements in critical ill patients without chronic respiratory disorders.

## Introduction

Mechanical ventilation (MV) is a life-saving intervention provided in over 20 million patients per year worldwide (1). It has been estimated that approximately 30% of patients admitted to the Intensive Care Unit (ICU) might require MV to support the patients’ breathing during critical illness (2–4). However, growing evidence highlighted that prolonged MV significantly affects functional outcomes and length of stay, with detrimental consequences in terms of residual disability and social and sanitary costs (3, 5–8). More in detail, prolonged MV has been related to physical and functional impairment, secretion clearance dysfunctions, respiratory, and skeletal muscles weakness, together with malnutrition, chronic cardiac and respiratory disease, depression, anxiety, and delirium (9).

Besides the above-mentioned physical sequelae, patients admitted to ICU requiring prolonged MV may consume more than 37% of ICU resources (10). Moreover, from 4 to 13% of ICU MV patients require more than 21 days of MV for at least 6 h/day, consuming 60% more healthcare resources than non-ventilated patients (11). Albeit MV might be considered mandatory in patients with acute respiratory failure, prolonged MV could cause several complications, including ventilator-associated pneumonia (VAP), lung infections and atelectasis (12). Furthermore, prolonged MV has been related to a higher risk of death in patients admitted to ICU (13).

Taken into consideration these aspects, a patient-tailored rehabilitation program aimed at optimizing weaning from MV should be considered as a cornerstone in the management of critically ICU patients to improve their physical and psychosocial outcomes (14).

Recent findings suggest that about 70% of ICU patients can be weaned successfully within the first day, while in 30% of cases the initial attempts fail with relevant negative implications in the weaning process (15). “Difficult-to-wean patients” requiring prolonged MV, account for up to 15% of those requiring MV in the ICU and weaning centers (16) and about 25% of them develop early muscle weakness (2).

To date, benefits from early mobilization, respiratory and physical therapy interventions have been supported in several conditions (17–21). Recently, a network meta-analysis (NMA), performed by Worraphan et al. (22), assessed the effectiveness of currently available physiotherapy interventions in facilitating weaning from MV. However, the authors focused only on inspiratory muscle training (IMT) and early mobilization (EM) interventions, while other rehabilitative strategies were not assessed (22). Similarly, the systematic review by Vorona et al. (23) assessed the effects of inspiratory muscle rehabilitation in critically ill adults reporting intriguing results in terms of safety and tolerability. However, the authors did not report specific indications about the precise rehabilitation program performed (23).

Despite the effects of physiotherapy interventions in weaning from MV have been deeply studied, to date, evidence in literature about the effects of different strategies is still lacking. Furthermore, to the best of our knowledge, no previous systematic review assessed the effects of different training programs in critical ill patients without chronic respiratory issues before the ICU admission.

In light of these considerations, strong evidence is needed to provide clinically relevant data to guide physicians in prescribing effective and safe physiotherapy interventions in order to improve the tailored rehabilitative management of prolonged MV critically ill patients.

Therefore, this systematic review of randomized controlled trials (RCTs) with meta-analysis aimed at summarizing the current evidence on the efficacy of targeted physiotherapy and/or comprehensive physiotherapy interventions to reduce MV duration and implement the weaning process in critically ill patients.

## Materials and Methods

### Registration

This systematic review of RCTs has been performed ethically in accordance with the Preferred Reporting Items for Systematic Reviews and Meta-analyses (PRISMA) statement (24). A protocol was developed before study initiation and submitted to PROSPERO^1^ (registration number CRD42022299537^2^).

### Search Strategy

We systematically searched PubMed/Medline, Scopus, and Web of Science for RCTs published up to October 22, 2021. Each source was searched on the same date. Two investigators independently searched the databases. The full search strategies for all databases are reported in Supplementary Table 1.

### Selection Criteria

In accordance with the PICO model (25), we considered eligible RCTs satisfying the following criteria:

– (P) Participants: acute patients admitted to ICU facilities and mechanical ventilated, age > 18 years, without pre-existent chronic respiratory conditions.
– (I) Intervention: we considered all rehabilitation and/or physiotherapy interventions identified by the search if they were protocolized (therapies were systematically provided to patients according to pre-defined algorithm or plan).
  More in detail, the rehabilitation treatments considered were:
  – *Positioning*, including all the changes in body positioning different from routine monotonic delivery of MV aiming at promoting the clearance of respiratory secretions, improving lung volume and oxygenation (26).
  – *Early mobilization techniques*, including active exercises or assisted exercises performed with patient’s own muscle strength occurred while the patient receive MV (27).
  – *ACTs and lung expansion*, including mechanical insufflation-exsufflation, percussion and vibrations, hyperinflation, and positive-expiratory-pressure devices (28).
  – *Respiratory muscle training*, including specific exercises aiming at improving respiratory muscle strength and function (29).
  – *Automatic Systems*, including mechanical support systems that automatically drive the level of pressure to promote and facilitate the discontinuation of MV through the early recognition of the patient’s ability to breathe spontaneously (30).
– (C) Comparator: any comparator;
– (O) Outcome: the primary outcomes were MV duration, extubation, and weaning time (defined as time between first assessment and the absence of MV for 48 h). The secondary outcomes were: (i) weaning successful rate (express as percentage of patients weaned per whole sample); (ii) changes in respiratory muscle or function (maximal inspiratory pressure, Tidal volume, respiratory muscle thickness); (iii) ICU discharge rate; (iv) ICU length of stay.

Only RCTs that were peer-reviewed and published in an International journal in English language were included.

The exclusion criteria were: (i) studies involving animals; (ii) participants with pregnancy, clinical instability, or palliation; (iii) Masters or doctorate theses and conference proceedings. No publication date restriction was applied.

After duplication removal, the remaining articles were screened by two investigators that independently reviewed the title and abstracts to choose relevant ones. Those that met all the inclusion criteria or that were ambiguous were kept for the second screening phase, which consisted of a full-text review. Any disagreements were discussed with a third reviewer to reach consensus. No automation tool was used in this process.

### Data Extraction and Synthesis

All the records screened in full-text were assessed for eligibility by two independent reviewers and relevant data were extracted through Excel. Any disagreement was solved by discussion between the two reviewers or consulting a third reviewer. No automation tool was used in the process.

The following data were extracted: (1) Authors; (2) Journal; (3) Publication year; (4) Nationality; (5) Population characteristics; (6) Intervention characteristics; (7) Comparator characteristics; (8) Outcomes; (9) Main findings.

A descriptive approach was used to synthesize both study characteristics and data extracted. The studies were grouped for the syntheses basing on the outcomes assessed.

Subgroup analysis has been performed based on the type of intervention proposed.

### Meta-Analysis

The meta-analysis was performed on Revman 5.4.0 (The Cochrane Collaboration, 2020, United States). The heterogeneity among comparisons was estimated by the chi-squared and I2 statistic tests. An I2 > 75% determined significant heterogeneity across the articles. In the event of considerable heterogeneity, a random-effects model was adopted to determine the pooled estimates with the effect size (ES) and 95% CIs. Missing means and SDs were estimated from medians, ranges, and interquartile ranges (IQRs) using the method introduced by Hozo et al. (31).

### Quality Assessment and Risk of Bias

The quality assessment was performed through PEDro scale by two independent reviewers. A third reviewer was involved in case of disagreement to achieve consensus. According to PEDro scale, the studies were rated as excellent (9–10 points), good (6–8 points), fair (4–5 points), or poor (<4 points).

The risk of bias was assessed through Version 2 of the Cochrane risk-of-bias tool for randomized trials (RoB 2) (32) by two reviewers independently. In case of disagreement, the consensus was achieved by discussion of consulting a third reviewer. Bias was reported by each domain of RoB 2 [(i) random sequence generation; (ii) allocation concealment; (iii) blinding of participants and personnel; (iv) blinding of outcome assessment; (v) incomplete outcome data; (vi) selective outcome reporting; (vii) other bias] and a rating (low, high, unclear) was assigned to each domain.

## Results

### Study Characteristics

Altogether, a total of 2503 records were identified from the 5 databases assessed. After duplication removal of 868 records, 1635 studies were assessed for eligibility and screened for title and abstract. As a result, 1543 records were excluded, and 92 studies were subsequently screened in full-text.

**FIGURE 1:**
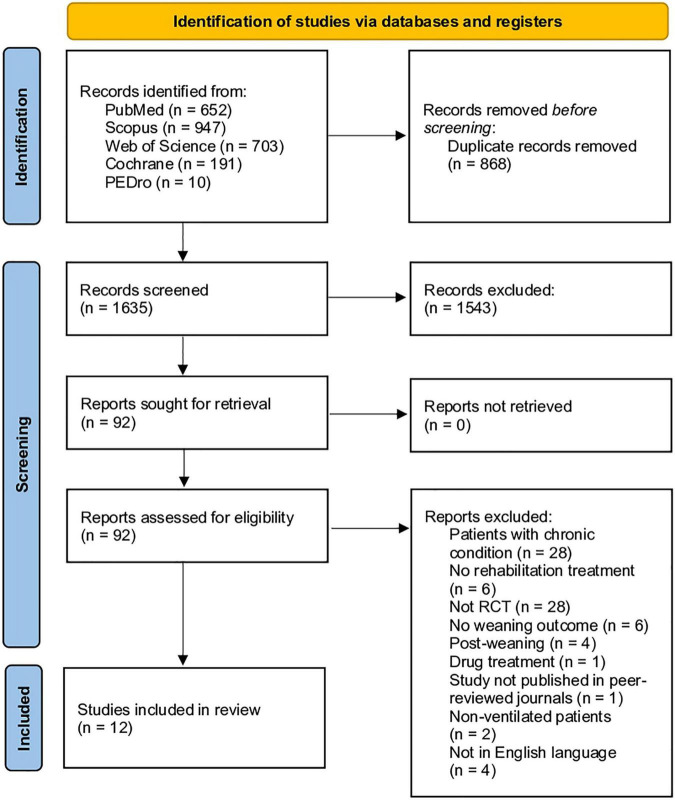
PRISMA 2020 flow chart.

Lastly, 12 RCTs (33–44) were included in the present systematic review (83 articles were excluded because not meet the eligibility criteria). Supplementary Table 1 shows the list of full-text studies reporting the reasons for exclusion. The PRISMA flow diagram reported the search process in detail (Figure 1).

Therefore, the following RCTs were included in the present systematic review: Berti (33), Cader (34), Cader (42), Chen (35), Dong (36), Liu and Zhang (43), Martin (37), McCaughey (38), Pattanshetty and Gaude (39), Pinkaew et al. (44), Sandoval Moreno (40), and Taniguchi (41).

The studies included were published between 2010 (39) and 2021 (36) and were conducted in America [United States n: 1 (37); Brazil *n* = 4 (33, 34, 41, 42); Colombia *n* = 1 (40)], Australia [*n* = 1 (38)], and Asia [China *n* = 2 (36, 43), India *n* = 1 (39); Taiwan *n* = 1 (35); Thailand *n* = 1 (44)].

The sample size of the RCTs included ranged from 20 (38) to 126 (40), for a total of 413 patients in the intervention groups (219 males and 194 females) and 378 in comparators (224 males and 154 females).

The subject assessed in the different studies were characterized by a mean age ranging from 47.8 ± 14.72 (39) to 83 ± 3 (34) in the intervention group, while mean age ranged between 47.85 (43) and 82 ± 7 (34) in the comparator group.

Interestingly, respiratory muscle training has been the physiotherapy intervention most studied in the RCTs included in the present systematic review (34, 37, 38, 40, 42, 43). On the other hand, three studies (36, 39, 40) assessed early mobilization/positioning, two studies (33, 35) assessed airway clearance techniques (ACTs) and lung expansion, and one study (41) assessed automatic systems for MV weaning.

The intervention groups were compared to standard care/physiotherapy (33–36, 39–44) or sham treatment (37, 38).

Table 1 summarizes in detail the sample characteristics of both intervention groups and comparator groups of each study included in the present review.

**TABLE 1:** Main characteristics of the studies included.

Authors and Publication year	Journal	Nationality	Population	Population Characteristics	Intervention	Comparator	Protocol duration	Main findings
Berti et al. (33)	*J Bras Pneumol.*	Brazil	ICU patients on MV IG: n: 16 CG: n: 19	IG: Mean age: 58.06 ± 13.81; Male/female:10/6 CG: Mean age: 55.42 ± 16.99; Male/female:13/6	MH (with a manual resuscitation bag, peak airway pressure of 40 cm H_2_O) and ERCC twice a day for 5 days, combined with standard nursing care.	Standard nursing care: positioning (changing the body position every 2 h throughout the day) and airway suctioning (was performed for four sets of six cycles, 15 s, six times a day)	5 days	This study reports about ICU patients on MV (mean age IG: 58.06 ± 13.81, CG: 55.42 ± 16.99; 63% male in IG, 68% male in CG). They were assessed for all 5 days of the protocol duration. The main finding is represented by the differences between groups in terms of patients weaned from MV (days 2: 0.0 vs. 37.5%; *p* < 0.01; days 3: 0.0 vs. 37.5%; *p* < 0.01; days 4: 5.3 vs. 37.5%; *p* < 0.05; days 5: 15.9 vs. 37.5%; *p* < 0.05). The ICU discharge rate was significantly higher in IG group (*p* < 0.05).
Cader et al. (34)	*J Physiother.*	Brazil	ICU patients on MV IG: n: 21 CG: n: 20	IG: Mean age: 83 ± 3; Male/female:9/12 CG: Mean age: 82 ± 7; Male/female:10/10	IMT, twice a day, 7 days a week, 5 min per session. The target regimen was to commence with a load of 30% of the participant’s maximal inspiratory pressure increasing daily by 10%.	Usual care	Weaning period (MV: IG: 3.6 ± 1.5 days; CG: 5.3 ± 1.9 days)	This study reports about ICU patients on MV (mean age IG: 83 ± 3; CG: 82 ± 7; 43% male in IG, 50% male in CG; mean weight IG: 66 ± 5; CG: 65 ± 6). They were assessed for all days of the protocol duration. The main finding is represented by the reduction of the weaning period by 1.7 days (95% CI 0.4 to 3.0) in ICU patients exposed to IMT (3.6 ± 1.5 days), as compared to usual care (5.3 ± 1.9 days). Maximal inspiratory pressure increased significantly in the IG than in the CG (MD 7.6 cmH20, 95% CI 5.8 to 9.4). The Tobin index decreased in both groups over the weaning period, with significant differences between groups (MD 8.3 br/min/L, 95% CI 2.9 to 13.7).
Cader et al. (42)	*Clin Interv Aging*	Brazil	ICU patients on MV IG: n: 14; CG: n: 14	IG: Mean age: 82 ± 4; Male/female: 6/8; CG: Mean age: 81 ± 6; Male/female: 7/7	Conventional physiotherapy + IMT with a threshold device: 5 min, twice daily, 7 days a week, with supplemental oxygen from the beginning of weaning until extubation.	Conventional physiotherapy	Weaning period (MV time: IG 10 days CG: 11 days)	This study reports about ICU patients on MV (mean age IG: 82 ± 4; CG: 81 ± 6; 42.86% of male in IG, 50,00% in CG). They were assessed 48 h after having undergone MV. The main finding is represented by the increase of maximum inspiratory pressure and significantly reduced the Tobin index. There was a significant and unsatisfactory increase in Tobin index for the control group (95% confidence interval [CI] −4.47 to −24.44, *p* = 0.002) and a satisfactory increase in maximum inspiratory pressure in the experimental group (95% CI 7.09–12.62, *p* = 0.001). The post-test indicated a significant reduction in Tobin index (95% CI −26.23 to −6.05, *p* = 0.001) and a significant increase in maximum inspiratory pressure (95% CI 4.67–10.19, *p* = 0.001) when compared to the control group. The authors did not find significant difference in extubation success (χ^2^ = 1.47; *p* = 0.20), although weaning duration was shorter in IG (3.64 ± 1.50 days) compared to CG (5.36 ± 1.87 days).
Chen et al. (35)	*Can Respir J.*	Taiwan	ICU patients on MV IPPB n: 17 IPPB + PEEP n: 16 CG: n: 17	IPPB: Mean age: 69.1 ± 11.1; Male/female: 7/10 IPPB + PEEP: Mean age: 76.4 ± 14.7; Male/female: 8/8 CG: Mean age: 72.3 ± 16.2; Male/female: 12/5	IPPB or IPPB + PEEP, target volume of 10–15 mL/kg of IBW, twice a day for 7 days, 20 min per session. Semi-Fowler’s position.	Usual care	8 days	This study reports about ICU patients on MV (mean age IPPB: 69.1 ± 11.1, IPPB + PEEP: 76.4 ± 14.7, CG: 72.3 ± 16.2; gender M/F IPPB: 10/7, IPPB + PEEP: 8/8, CG: 5/12; mean weight IPPB: 56.9 ± 12.0, IPPB + PEEP: 57.3 ± 14.5, CG: 59.4 ± 12.0). The main finding is represented by the significantly higher weaning rate in IPPB and IPPB + PEEP groups compared to control group (IPPB vs. IPPB + PEEP vs. control: 88.2 vs. 87.5 vs. 41.2%, *p* < 0.05). Patients in the IPPB group showed a significant increase in Tindal Volume after 7 days (pre vs. post: 240.4 ± 57.2 vs. 292.5 ± 116.3 mL, *p* < 0.05), while the control group showed a significant reduction (pre vs. post: 293.3 ± 168.9 mL vs. 243.9 ± 140.4 mL, *p* < 0.05). In the IPPB + PEEP group, a significant increase in MIP was observed after the intervention (29.9 ± 15.0 vs. 37.0 ± 16.5 cmH_2_O, *p* < 0.05).
Dong et al. (36)	*BMC Pulm Med*	China	ICU patients on MV IG: n: 39 CG: n: 41	IG: Mean age: 59.05 ± 17.61; Male/female: 25/14 CG: Mean age: 64.44 ± 14.72; Male/female: 23/18	Early rehabilitation therapy: six levels of rehabilitation exercises, from positioning and rotational therapy to walking near the bedside.	Standard care	3–4 days	This study reports about ICU patients on MV (mean age IG: 59.05 ± 17.6; CG: 64.44 ± 14.72; 64.10% male in IG, 56.10% male in CG; mean BMI IG: 23.18 ± 3.32; CG: 23.22 ± 3.67). They were assessed for diaphragmatic excursion and diaphragmatic thickening fraction at 1- and 4-day of MV. The main finding is represented by the improvement observed in MV duration (7.49 ± 2.59 days vs. 9.41 ± 5.32 days; *p* = 0.045) and a significantly shorter intubation duration (8.31 ± 2.80 days vs. 10.37 ± 5.32 days, *p* = 0.037). The two groups were comparable in terms of duration of ICU stay (*p* = NS). At 4-day MV, the IG had significantly decreased diaphragmatic thickening fraction compared to the control group (0.15 ± 0.06 g vs. 0.12 ± 0.05 g, *p* = 0.008).
Liu and Zhang (43)	*Indian J Pharm Sci*	China	ICU patients on MV IG: n: 50 CG: n: 50	IG: Mean age: 48.07 (range: 32–70); Male/female:26/24; CG: Mean age: 47.85 (range: 30–70); Male/female:24/26	Artificial airway humidification is performed every 1∼2 h. The patient’s position is changed every 2 h, turning over and knocking back to help the patient to perform active or passive joint movement. A transcutaneous electrical nerve stimulator is used to perform neuromuscular electrical stimulation. A suitable sandbag has been positioned on the belly area to exercise his respiratory muscles.	Nursing inspection 3 times a day. Symptomatic treatment may be proceeded, such as relieve cough resolve phlegm, medication, transfusion therapy and diet care, etc.	Until ICU discharge (ICU length of stay) IG: 10.47 ± 2.55 CG: 18.84 ± 5.37	This study reports about ICU patients on MV (mean age IG: 48.07 [range: 32–70]; CG: 47.85 [range: 30–70]; 50% male). They were assessed for all days of the protocol duration. The main finding is represented by the success rate of weaning in the IG which was 92.0% (*p* < 0.05). The incidence of VAP in the IG was significantly lower (8.0%) than that in the CG (34.0%), and the difference was statistically significant (*p* < 0.05). The average MV time of the CG was 13.54 ± 4.75 days, and the average MV time of the IG was 6.14 ± 2.07 days (*p* < 0.05). The average length of stay was 18.84 ± 5.37 days stay in the CG, while in the IG was 10.47 ± 2.55 days (*p* < 0.05). Compared to before treatment, the CPIS of the two groups was significantly lower after treatment (*p* < 0.05), and the IG was significantly lower than the CG, the difference was statistically significant (*p* < 0.05).
Martin et al. (37)	Critical Care	United States	ICU patients on MV IG: n: 35 CG: n: 34	IG: Mean age: 65.6 ± 11.7; Male/female: 16/19 CG: Mean age: 65.1 ± 10.7; Male/female: 15/19	IMT: 5 days per week with a threshold inspiratory muscle training (pressure load between −4 and −20 cmH_2_O)	Sham treatment with a resistive inspiratory muscle training device	Weaning period (Total study days: IG: 14.4 ± 8.1 days; CG: 18.0 ± 8.8 days)	This study reports about ICU patients on MV (mean age IG: 65.6 ± 11.7, CG: 65.1 ± 10.7; gender M/F: 16/19 in IG, 15/19 in CG). They were assessed on the first day of participation, every Monday and on days when the subjects attempted a 12-h aerosol tracheotomy collar (ATC) trial. The main finding is represented by the improvement observed in maximal inspiratory pressure (MIP) where the sham group’s pre- to post-training MIP change was not significant (−43.5 ± 17.8 vs. −45.1 ± 19.5 cm H_2_O, *p* = 0.39), while the IMT group’s MIP increased (−44.4 ± 18.4 vs. −54.1 ± 17.8 cm H_2_O, *p* < 0.0001). There were no adverse events observed during IMT or sham treatments. Twenty-five of 35 IMT subjects weaned (71, 95% confidence interval (CI) = 55 to 84%), while 16 of 34 (47, 95% CI = 31 to 63%) sham subjects weaned. The pre- and post-training MIP measures for the weaning success (*n* = 41) and failure (*n* = 28) groups were respectively (−44.0 ± 20.2 and −53.5 ± 20.7 cmH_2_O vs. −43.9 ± 14.8 and −43.9 ± 15.0 cmH_2_O). There was significant outcome × time interaction and the change in MIP for the successfully weaned group was significantly greater than the failure to wean group (*p* < 0.0001).
McCaughey et al. (38)	*Critical Care*	Australia	ICU patients on MV IG: n: 10 CG: n: 10	IG: Mean age: 56.5 ± 18.50 [median ± (IQR)]; Male/female: 7/3 CG: Mean age 61.0 ± 17.25 [median ± IQR)]; Male/female:5/5	Active abdominal FES training, 30 min, twice per day, 5 days per week. FES was set to a median of 60 mA, frequency of 30 Hz and a pulsewidth of 350 μs;	Sham abdominal FES training. FES was set to a median of 10 mA, frequency of 10 Hz and a pulsewidth of 350 μs.	Until ICU discharge (ICU length of stay) IG: not estimable; CG: 11 days;	This study reports about ICU patients on MV (mean IG: 56.5 (IQR 18.50), CG: 61.0 (IQR 17.25); gender M/F: 7/3 in IG, 5/5 in CG). They were assessed twice more in the first week of participation, and then weekly until ICU discharge. The main finding is represented by the improvement observed in ventilation duration (median 6.5 vs. 34 days, *p* = 0.039) and ICU length of stay (median 11 vs. not estimable days, *p* = 0.011) that were shorter in IG compared to the control group. However, no significant differences were underlined in terms of muscle thickness in rectus abdominis (*p* = 0.099 at day 3), diaphragm (*p* = 0.652 at day 3) or combined lateral abdominal muscles (*p* = 0.074 at day 3). The authors were unable to adequately assess MIP due to tracheostomy.
Pattanshetty and Gaude (39)	*Indian J Crit Care Med*	India	ICU patients on MV IG: n:50 CG: n:51	IG: Mean age: 47.8 ± 14.72; Male/female: 37/13 CG: Mean age: 51.6 ± 17.47; Male/female: 40/11	Positioning + chest wall vibrations + MH + suctioning MH: daily, twice a day, 20 min per session; Chest vibration: daily, twice a day, thrice in each zone (upper, middle, lower of chest); Suctioning: once every minute for 4 min, 15 s	MH + suctioning	Weaning period (MV: IG: 13.9 ± 9.77; CG: 11.3 ± 5.73)	This study reports about ICU patients on MV (mean age IG: 47.8 ± 14.72, CG: 51.6 ± 17.47; gender M/F: 37/13 in IG, 40/11 in CG). They were assessed after 48H from MV during the weaning period, before and after physiotherapy. The main finding is represented by the improvement observed in weaning of ventilation, successful in the case of 62% of the patients in the IG as compared to 31.37% of the patients in the CG, which was statistically significant (*p* = 0.007).
Pinkaew et al. (44)	*Indian J Public Health Res Dev*	Thailand	ICU and Sub-ICU patients on MV EMEB: n: 25 EM: n: 23 CG: n: 23	EMEB group: Mean age: 75.32 ± 14.28; Male/female: 7/18 EM group: Mean age: 69.08 ± 16.96; Male/female: 11/12 CG: Mean age: 74.68 ± 15.23; Male/female: 15/8	EMEB: Traditional therapy + EM protocol + elastic exercise in diagonal pull, shoulder flexion, flyer and reverse flyer postures, 10 times 3 sets, once a day, 5 times a week. The group took about 30 min to treat each time and provided treatment 5 days a week. EM: Traditional therapy + EM protocol (4 levels = level 1 is passive ROM; level 2 is passive ROM, active ROM, and sitting position minimum 20 min; level 3 is passive ROM, active ROM, sitting position minimum 20 min and sitting on edge of bed; level 4 is passive ROM, active ROM, sitting position minimum 20 min, sitting on edge of bed, active transfer to chair minimum 20 min)	Conventional physical therapy groups included passive and active ROM, breathing exercise, 5 times a week	Weaning period (MV time: EM: 5.78 ± 2.74 EMEB: 6.52 ± 4.40 CG: 12.82 ± 5.69)	This study reports about ICU patients on MV (mean age CG: 74.68 ± 15.23; EM: 69.08 ± 16.96; EMEB: 75.32 ± 14.28; 33 male and 38 female). They were assessed for all days of the protocol duration. The main finding is represented by the significant differences of MV duration (days) between the CG, the EM group and the EMEB group, that were 12.82 ± 5.69, 5.78 ± 2.74 (*p* < 0.05) and 6.52 ± 4.40 (*p* < 0.05), respectively. EMEB showed significantly increased handgrip strength changes compared to CG (IG: 3.53 ± 1.42 kgs; CG: 0.97 ± 1.21kgs; *p* < 0.05).
Sandoval Moreno et al. (40)	*Med Intensiva*	Colombia	ICU patients on MV IG: n: 62 CG: n: 64	IG: Mean age: 61 (range: 40–70); Male/female: 33/29 CG: Mean age: 62 (range: 47–72); Male/female: 38/26	Respiratory muscle training with threshold IMT, every day, twice a day for 3 series of 6–10 repetitions, with 2 min of rest between series.	standard care: respiratory physiotherapy, physical therapy, and MV management	Weaning period (MV time: IG:9.36 ± 12.51; CG: 8.78 ± 11.41)	This study reports about ICU patients on MV (mean age IG: 61 (range: 40–70), CG: 62 (range: 47–72); 53.23% male in IG, 59.38% male in CG). They were assessed after 48H from MV during the weaning period. There were no statistically significant differences in the median weaning time between the groups. There were no statistically significant differences in the median change in MIP between the groups (IG: 9.43 cmH_2_O vs. CG: 5.92 cmH_2_O; *p* = 0.48).
Taniguchi et al. (41)	*Critical Care*	Brazil	ICU patients on MV IG: n: 35 CG: n: 35	IG: Mean age: 66 ± 18; Male/female: 17/18 CG: Mean age: 62 ± 19; Male/female: 22/13	SmartCare device (the ventilator automatically adjusted pressure support at the minimum level while keeping the patient within a comfort zone)	Respiratory physiotherapy consisting of breathing spontaneously through PSV of 5–7 cmH_2_O and PEEP of 5 cmH_2_O, for a minimum of 30 min and a maximum of 2 h.	Weaning period (MV time: IG: 3.5 (2.0–7.3) CG: 4.1 (2.7–7.1)	This study reports about ICU patients on MV (mean age IG: 66 ± 18 (range: 20–93); CG: 62 ± 19 (range: 33–97); 49% male in IG, 63% male in CG). They were assessed during the weaning period. The main finding is represented by the improvement observed in weaning duration, which was shorter in the respiratory physiotherapy–driven weaning group (60 [50–80] min vs. 110 [80–130] min; *p* < 0.001). Total duration of MV (3.5 [2.0–7.3] days vs. 4.1 [2.7–7.1] days; *p* = 0.467) and extubation failure (2 vs. 2; *p* = 1.00) were similar between the two groups. No significant differences between groups were underlined in Tidal Volume.

*Continuous variables are expressed as means ± SD, unless otherwise stated. CI, confidence interval; CG, control group; EM, early mobilization; EMEB, early mobilization with elastic band; ERCC, expiratory rib cage compression; FES, functional electrical stimulation; ICU, intensive care unit; IG, intervention group; IMT, inspiratory muscle training; IPPB, intermittent positive pressure breathing; IQR, interquartile range; MD, mean differences; MH, manual hyperinflation; MIP, maximal inspiratory pressure; MV, mechanical ventilation; PEEP, positive end-expiratory pressure; PSV, pressure support ventilation; ROM, range of motion; USA, United States of America.*

### Intervention Characteristics

Rehabilitation treatments have been classified as early mobilization, positioning, ACTs/lung expansion, respiratory muscle training and automatic weaning systems.

– *Positioning and early mobilization*: three studies (36, 39, 44) assessed the effects of different positioning and early mobilization programs. In particular, Dong et al. (36) assessed a progressive rehabilitation program composed of six levels of intensity, from positioning and rotational therapy to walking near the bedside. The protocol duration varied between 3 and 4 days.

Differently, Pattanshetty and Gaude (39) assessed the effects of a comprehensive rehabilitation intervention including positioning, in addition to chest wall vibrations, manual hyperinflation (MH), and suctioning. The rehabilitation program was proposed for 20 min twice a day.

Lastly, Pinkaew et al. (44) assessed the effects of two different groups compared to conventional treatment. More in detail, one interventional arm received traditional therapy, early mobilization, and exercises with elastic band for 3 sets of 10 repetitions once a day, 5 times a week. On the other hand, the second interventional group was treated only with conventional therapy and early mobilization.

– *ACTs and lung expansion*: two studies (33, 35) assessed ACTs and lung expansion strategies. More in detail, Berti et al. (33) assessed the effects of manual hyperinflations (peak airway pressure of 40 cmH_2_O) combined with chest compression twice a day for 5 days. In contrast, Chen et al. (35) assessed the role of two different rehabilitation programs assessing intermittent positive pressure breathing (IPPB) alone or combined with positive end-expiratory pressure (PEEP) (target volume of 10–15 mL/kg of IBW), twice a day for 7 days, 20 min per session.
– *Respiratory muscle training:* six studies (34, 35, 38, 40, 42, 43) assessed respiratory muscle training proposing different therapeutic strategies. In particular, Cader et al. (34) in 2010 assessed the effect of an IMT protocol twice a day, 7 days a week, 5 min per session. The protocol was characterized by a load of 30% of the participant’s maximal inspiratory pressure increasing daily by 10%. In 2012, the same working group (42) assessed the effects of conventional physiotherapy combined with IMT with a threshold device and the same protocol used in 2010.

Similarly, Martin et al. (37) assessed the effect of an IMT protocol 5 days per week with a threshold inspiratory muscle training device (pressure load between −4 and −20 cmH_2_O). According to Sandoval Moreno et al. (40) respiratory muscle training was performed with threshold IMT respiratory muscle trainer, every day, twice a day for 3 series of 6–10 repetitions, with 2 min of rest between series.

Interestingly, McCaughey et al. (38) combined conventional therapy with abdominal functional electrical stimulation (FES) (38), 30 min, twice per day, 5 days per week. FES was regulated to a median of 60 mA, frequency of 30 Hz, and a pulsewidth of 350 μs.

Lastly, Liu and Zhang (43) assessed the effects of a comprehensive rehabilitation treatment including positioning and active or passive joint movement, combined with neuromuscular electrical stimulation and respiratory muscle training with a suitable sandbag positioned on the belly area.

– *Automatic Systems*: only one study (41) assessed the role of an automated weaning program SmartCare™ included in a mechanical ventilator that automatically adjusted the pressure support at the minimum level, while keeping the patient within a comfort zone.

All the rehabilitation programs of the RCTs assessed in the present systematic review have been summarized in Table 1.

### Main Findings – Weaning Duration, Extubation, and Weaning Time

Altogether, 9 RCTs (34–36, 38, 40–44) assessed weaning duration. In particular, Dong et al. (36) showed advantages in positioning and early rehabilitation (ER) group, underlining significantly shorter duration of ventilator use (7.49 ± 2.59 days vs. 9.41 ± 5.32 days; *p* = 0.045) and a significantly shorter duration of intubation (8.31 ± 2.80 days vs. 10.37 ± 5.32 days; *p* = 0.037) compared to standard care. Accordingly, Pinkaew et al. (44) showed that the duration of MV of the CG, the ER group and the ER with elastic band group were respectively 12.82 ± 5.69 days, 5.78 ± 2.74 (*p* < 0.05) days, and 6.52 ± 4.40 days (*p* < 0.05).

Interestingly, Chen et al. (35) that assessed ACTs and lung expansion, underlined significant differences of MV days between IPPB alone and combined with PEEP when compared to control group (11.7 ± 3.7 days vs. 15.8 ± 9.1 days vs. 27.2 ± 16.1 days respectively; *p* < 0.05).

On the other hand, respiratory muscle training was assessed by Cader et al. (34) that reported a significant reduction of the weaning period by 1.7 days (95% Confidence Interval (CI): 0.4 to 3.0) in ICU patients treated with IMT (3.6 ± 1.5 days) compared to usual care (5.3 ± 1.9 days). Accordingly, the same authors in 2012 (42) reported shorter weaning times in the experimental group (3.64 ± 1.50 days) compared to the CG (5.36 ± 1.87 days). Concurrently, McCaughey et al. (38) reported a significant improvement in ventilation duration (median 6.5 vs. 34 days; *p* = 0.039) after active abdominal FES training compared to the control group. Similarly, Liu and Zhang (43) assessed a comprehensive rehabilitation treatment reporting significant differences between groups in terms of MV duration (6.14 ± 2.07 vs. 13.54 ± 4.75 days; *p* < 0.05). In contrast, Sandoval Moreno et al. (40) when assessing the effects of respiratory muscle training found no statistically significant differences in median weaning time between groups (8.78 ± 11.41 h vs. 9.36 ± 12.51 h; *p* = NS).

Lastly, Taniguchi et al. (41) in assessing the automated system for MV weaning reported a shorter weaning duration in the respiratory physiotherapy–driven weaning control group (60 [50–80] min vs. 110 [80–130] min; *p* < 0.001). Total duration of mechanical ventilation was 3.5 [2.0–7.3] days in physiotherapy–driven weaning group, compared to 4.1 [2.7–7.1] days in the automated system group (*p* = 0.467).

### Main Findings – Weaning Successful Rate

Out of the 12 studies included, 8 RCTs (33, 35, 37, 39–43) assessed weaning successful rate. More in detail, Pattanshetty and Gaude (39) reported a significant improvement (*p* = 0.007) in weaning successful rate after a comprehensive rehabilitative intervention including positioning, chest wall vibrations, MH, and suctioning. The intervention was successful in 62% of the patients in the positioning and ER group compared to 31.37% of the patients in the CG.

On the other hand, Berti et al. (33) reported significant benefit after an ACT and lung expansion intervention at different timepoints (IG: days 2: 0.0 vs. 37.5%; *p* < 0.01; days 3: 0.0% vs. CG: 37.5%; *p* < 0.01; days 4: 5.3 vs. 37.5%; *p* < 0.05; days 5: 15.9 vs. 37.5%; *p* < 0.05). Similarly, Chen et al. (35) reported a significantly higher weaning rate in IPPB and IPPB + PEEP groups compared to control group (IPPB vs. IPPB + PEEP vs. control: 88.2 vs. 87.5 vs. 41.2%, *p* < 0.05).

Weaning successful rate after respiratory muscle training intervention was assessed by Martin et al. (37), reporting that 25 of 35 of patients undergoing respiratory muscle training are successfully weaned from MV (71, 95% CI = 55 to 84%), in contrast with 16 of 34 subjects of the CG (47, 95% CI = 31 to 63%). Accordingly, Liu and Zhang (43) reported a weaning success rate of 92.0% after a comprehensive rehabilitation program, underlining significant differences between groups (*p* < 0.05).

On the contrary, Cader et al. (42) highlighted no significant differences in extubation success between the groups (*p* = 0.20). Similarly, Sandoval Moreno et al. (40) showed that weaning successful rate was 75.81% in the rehabilitation group while it was 75% in the control group, with no significant differences between groups (*p* = NS).

Finally, Taniguchi et al. (41) reported that extubation failure (2 vs. 2; *p* = 1.00) was similar between the automated system weaning group and physiotherapy–driven weaning group.

Table 1 summarizes the main findings of the RCTs included in the present systematic review.

### Main Findings – Respiratory Function

Maximum inspiratory pressure (MIP) represented the most common respiratory function parameter assessed by the RCTs included in the present review (34, 35, 37, 38, 40, 41). More in detail, Chen et al. (35) reported a significant increase in MIP after lung expansion rehabilitation with IPPB combined with PEEP (29.9 ± 15.0 vs. 37.0 ± 16.5 cmH_2_O; *p* < 0.05).

Concerning respiratory muscle training, Cader et al. (34) reported a significant improvement of MIP after respiratory training program compared to control group (7.6 cmH_2_O, 95% CI 5.8 to 9.4). Accordingly, in 2012 the same authors (42) reported a significant increase in maximum inspiratory pressure (95% CI 4.67–10.19; *p* = 0.001) in IMT group when compared to the control group. Similarly, Martin et al. (37) reported a significant increase in MIP (−44.4 ± 18.4 vs. −54.1 ± 17.8 cmH_2_O; *p* < 0.0001) in IMT group, in contrast to sham control group (−43.5 ± 17.8 vs. −45.1 ± 19.5 cmH_2_O; *p* = 0.39). On the other hand, Sandoval Moreno et al. (40) did not find statistically significant differences in the median change in MIP between the groups (IG: 9.43 cmH2O vs. CG: 5.92 cmH_2_O; *p* = 0.48) after the IMT rehabilitation protocol. Lastly, McCaughey et al. (38) were unable to adequately assess MIP due to tracheostomy.

Differently, Tobin index has been assessed by 2 studies. In particular, Cader et al. (34) in 2010 assessed showed significant differences between groups in Tobin index after the respiratory muscle training program (8.3 br/min/L, 95% CI 2.9 to 13.7). Accordingly, in 2012 the same working group (42) reported a significant reduction in Tobin index (95% CI −26.23 to −6.05; *p* = 0.001).

Tidal volume has been assessed in 2 RCTs (35, 41). More in detail, Chen et al. (35) showed a significant increase in Tidal Volume after 7 days (pre vs. post: 240.4 ± 57.2 vs. 292.5 ± 116.3 mL; *p* < 0.05) in patients undergoing IPPB rehabilitation.

Instead, Taniguchi et al. (41) assessed tidal volume without underlining significant differences between groups comparing automated system weaning group to physiotherapy–driven weaning protocol.

Respiratory muscle thickness have been assessed by 2 RCTs (36, 38). In particular, Dong et al. (36) underlined significant differences between groups in diaphragmatic thickening fraction (IG: 0.15 ± 0.06 g vs. CG: 0.12 ± 0.05 g; *p* = 0.008) after progressive ER intervention. On the other hand, McCaughey et al. (38) reported no significant differences in rectus abdominis (*p* = 0.099), diaphragm (*p* = 0.652), or combined lateral abdominal muscles (*p* = 0.074) after 3 days of active abdominal FES training. See Table 1 for further details.

### Main Findings – Intensive Care Unit Discharge Rate and Length of Stay

Only one study (33) assessed discharge rate, reporting that ICU discharge rate was significantly higher in IG group (*p* < 0.05) after the rehabilitation treatment including ACTs and lung expansion.

In contrast, length of stay has been assessed in 4 studies (35, 36, 38, 43). In particular, Dong et al. (36) did not underline significant differences in terms of length of stay in ICU. Accordingly, Chen et al. (35) failed to show significant differences in terms of length of stay (IPPB vs. IPPB + PEEP vs. CG: 24.9 ± 10.7 vs. 23.6 ± 8.6 vs. 31.2 ± 13.1; *p* = NS).

In contrast, McCaughey et al. (38) reported a significant improvement in length of stay in ICU (median 11 vs. not estimable days; *p* = 0.011) after active abdominal FES training compared to the control group. Liu and Zhang (43) reported an average length of stay of 18.84 ± 5.37 days stay in the CG, while in the IG treated with a comprehensive rehabilitation program was 10.47 ± 2.55 days (*p* < 0.05). Table 1 shows the main findings of the studies included in detail.

### Meta-Analysis

**FIGURE 2:**
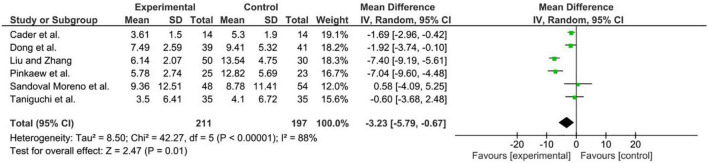
Results of our meta-analysis.

A meta-analysis was performed to highlight the efficacy of rehabilitative interventions on weaning duration in mechanically ventilated critically ill patients, showing an overall ES of −3.23 days (95% CI = −5.79, −0.67, *p* = 0.01; *Z* = 2.47) in decreasing MV time. Given the low number of RCTs, and the high heterogeneity a random-effects model was adopted (for further details see Figure 2).

### Study Quality

The quality assessment performed according to PEDro scale classified 9 studies (75%) (34–42, 44) as being of good quality and 3 studies (25%) (33, 36, 42) as being of fair quality. Table 2 shows the results of the study quality assessment by reporting the score of each subitem of the PEDro scale.

**TABLE 2:** Quality assessment of the studies included in the present systematic review according to the PEDro scale.

*Articles*	*Criteria for the quality scoring*										*Score*	*Quality level*
	*1*	*2*	*3*	*4*	*5*	*6*	*7*	*8*	*9*	*10*		
Berti et al. (33)	1	1	1	0	0	0	0	0	1	1	5	Fair quality
Cader et al. (34)	1	1	1	0	0	0	0	1	1	1	6	Good quality
Cader et al. (42)	1	1	1	0	0	0	0	0	1	1	5	Fair quality
Chen et al. (35)	1	1	1	0	0	0	1	0	1	1	6	Good quality
Dong et al. (36)	1	0	1	0	0	0	1	0	1	1	5	Fair quality
Liu and Zhang (43)	1	1	1	0	0	0	1	1	1	1	7	Good quality
Martin et al. (37)	1	1	1	1	0	0	1	1	1	1	8	Good quality
McCaughey et al. (38)	1	1	1	0	0	1	1	1	1	1	8	Good quality
Pattanshetty and Gaude (39)	1	1	1	0	0	0	1	0	1	1	6	Good quality
Pinkaew et al. (44)	1	1	1	0	0	0	1	1	1	1	7	Good quality
Sandoval-Moreno et al. (40)	1	1	1	0	0	1	1	1	1	1	8	Good quality
Taniguchi et al. (41)	1	1	1	0	0	0	1	1	1	1	7	Good quality

**FIGURE 3:**
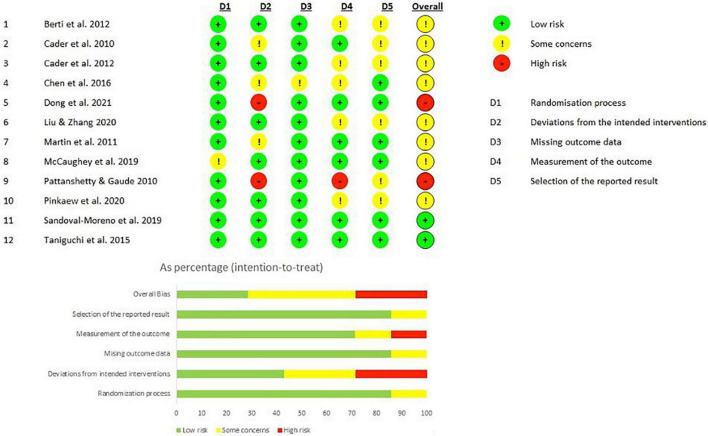
Risk of bias of the included studies according to the RoB2.

The assessment of the risk of bias was performed by RoBv.2 (32), highlighting that 11 studies (91.6%) (33–37, 39–44) were characterized by a low risk of bias in the randomization process, while 1 study (8.3) (38) showed some concerns. Two studies (16.7%) (36, 39) showed high risk of bias in the second domain, 3 studies (25%) (34, 35, 37) showed some concerns, and 7 studies (58.3%) (33, 38, 40–44) showed low risk of bias. Eleven studies (91.6%) (33, 34, 36–44) showed a low risk of bias in missing outcome data and just one study (34) showed some concerns. Six studies (50%) (34, 36–38, 40, 41) showed a low risk of bias in the fourth domain and 6 studies (50%) (34–37, 40, 41) showed a low risk of bias in the fifth domain. The overall risk of bias underlined two studies (16.7%) (40, 41) with low risk of bias, 8 studies (66.7%) (33–35, 37–39, 42–44) with some concerns, and 2 studies (16.7%) (36, 39) with a high risk of bias. Figure 3 shows the score of each subitem of RoBv.2 in detail.

## Discussion

Rehabilitation is widely considered a cornerstone in the complex process of weaning from MV (45, 46). However, to date, specific rehabilitative indications and precise strategies are still lacking, despite current research is now focusing on tailored rehabilitative programs aimed at reducing weaning duration and improving weaning successful rate (47). In this scenario, this systematic review of RCTs summarized the available evidence in literature targeting specific rehabilitative strategies that could represent a valid therapeutical approach to improve the weaning process and reduce MV duration in critically ill patients.

To date, growing attention has been placed on a combined rehabilitative approach for weaning in MV patients in order to improve the synergism between different therapeutic interventions (22, 48).

Noticeably, the results of our meta-analysis underlined the efficacy of a comprehensive rehabilitation intervention in reducing MV duration [ES: −3.23 days (95% CI = −5.79, −0.67, *p* = 0.01; *Z* = 2.47)]. These results suggest the key role of a specific pulmonary physiotherapy intervention in the ICU setting in order to minimize MV complications and optimize the functional recovery in critically ill patients. Similarly, the recent systematic review by Worraphan et al. (22) underlined significant improvement in weaning duration after EM and IMT combined with conventional physiotherapy. On the other hand, the authors assessed RCTs including patients suffering from chronic respiratory diseases with potential muscle impairment related to other pathological conditions that might significantly affect the treatment outcomes (22).

Concurrently, physical rehabilitation interventions have been otherwise defined in literature as ‘conventional physiotherapy,’ ‘usual therapy,’ ‘rehabilitation activity, ‘early mobilization,’ ‘respiratory physiotherapy,’ without deeply characterizing rehabilitative protocol or omitting the description of single interventions (33–44, 49, 50). In this scenario, activities such as passive and active limbs exercises, positioning, change of postures in bed, sitting on the edge of the bed, neuromuscular electrical stimulation (NMES) of peripheral muscles, secretion management and lung recruitment strategies can be integrated into the above definitions (33–44, 49, 50). As a result, a large gap of knowledge has been identified in the current literature, highlighting that only few articles (36, 43, 44) deeply characterized the components of EM protocols.

Therefore, we focused on a specific characterization of four main rehabilitative strategies that were categorized as early mobilization, ACTs including lung recruitment/expansion components, respiratory muscle training, and automated systems (33–44). Interestingly, positioning and early mobilization proved to be safe interventions for critically ill patients during weaning from MV, with recent evidence suggesting positive effects on weaning time and ICU length of stay (51, 52). Although evidence is still debated, ER protocols are recommended and widely employed to prevent or mitigate the ICU-acquired weakness and to improve clinical outcomes in acutely hospitalized adults who have been mechanically ventilated for more than 24 h (53).

On the other hand, albeit rehabilitation interventions in critical ill MV patients have been focused mainly on positioning and peripheral muscle training (23, 54), a growing interest has been raising in IMT in recent years (14). To date, IMT is a well-known effect of prolonged MV, and there is growing evidence that specific IMT can improve strength and endurance or reduce ventilator-induced diaphragm weakness (55). The systematic review and meta-analysis by Vorona et al. (23), underlined that IMT is a feasible and safe intervention in MV patients, suggesting IMT as a potential key component of an integrated rehabilitation program in difficult-to-wean patients with diaphragmatic weakness (23). However, the authors included only chronic patients in this review, with significant implications in terms of study results, given the chronic muscle alterations induced by respiratory conditions and the modifications in secretion production (23). On the other hand, a precise patient’s stratification is the cornerstone of specific physiotherapy interventions tailored to patient’s characteristics (56). Accordingly, the present systematic review and meta-analysis includes acute critical patients with no previous chronic respiratory conditions that might have affected respiratory mechanics or the potential respiratory muscles response to training (57–59). Our findings emphasize the positive contribution of specific IMT strategies using a threshold load that deeply characterizes the rehabilitation strategies inducing specific results on lung function (34, 37, 42). Despite conflicting results were reported by the RCTs included in the present work (40), IMT might represent a suitable option in ICU patients during weaning period, especially in patients with a proven IM weakness. In particular, Martin et al. (37) reported a significant improvement of MIP and less weaning time for patients trained with IMT and observed that successfully weaned patients had a significantly greater change in MIP than those in fail-to-wean group, even if trained with the IMT protocol (37). This interesting information suggests that the rehabilitative effort should be targeted to patients with a proven IM weakness. Therefore, it is important to monitor daily changes to identify the optimal responders to better focus on then rehabilitation efforts. On the other hand, although IMT has proven to be a safe treatment, it should be noted that the risk for exercise-induced muscle damage should be considered in severely debilitated patients (60). Interestingly, the systematic review from Elkins et al. (14) previously assessed the role of IMT in facilitating weaning from MV among patients in ICU. The authors reported positive results of IMT in selected patients (14). However, no previous systematic review assessed the role of IMT in a comprehensive rehabilitative approach. Moreover, to the best of our knowledge, the present work is the first systematic review and meta-analysis assessing acute ICU patients without chronic respiratory conditions, targeting a specific population in order to promote a specific therapeutical approach. Interestingly, the results of our quantitative synthesis have shown the efficacy of a comprehensive rehabilitation intervention including IMT in patients in ICU.

Despite these findings, the optimal parameters for IMT are yet to be established. However, it has been proposed that treatment intensity should be prescribed based on maximum inspiratory pressure and, if conditions do not allow to measure MIP, could be titrated *via* a trial-and-error method, starting from a low resistance, and gradually increasing intensity based on tolerance, symptoms and changes in vital parameters (61).

Moreover, expiratory muscle strength is currently considered an independent predictor of weaning success (62) and transversus abdominis, internal and external obliques muscle plays a crucial role in protecting airways with cough (63). Unfortunately, abdominal FES assessed by McCaughey et al. (38) did not show significant changes in terms of muscle strength. However, abdominal FES might be considered as a feasible rehabilitative option in patients with low compliance levels to other rehabilitation strategies.

On the other hand, ACTs and lung expansion strategies have been suggested as key components of rehabilitation interventions aimed at keeping the airways patency and reducing the work of breathing (64). While most of the studies included in the present review assessed different strategies (including suctioning, posture changes, percussion, and vibration), only two studies (33, 35) investigated the effects of different techniques on the weaning process. Our findings suggested positive effects of ACTs intervention on weaning duration. However, it should be noted that ACTs are prone to many confounding factors and the effects of this specific rehabilitation intervention alone have not been widely investigated. On the other hand, it has been reported that the use of PEEP can increase the functional residual capacity, keeping the alveoli and airways open during the expiratory phase, with positive implications in work of breathing and clearance of secretion (65).

Interestingly, while IPPB alone had the best weaning rate, IPPB + PEEP seems to have better effects on sputum production in the first session and better inspiratory muscle strength at the end of the study (35). However, further investigations are needed to clarify the promising contribution of these two strategies to success of first spontaneous breathing trials. In this scenario, Pattanshetty et al. (39) investigated the impact of positioning and chest wall vibrations over manual hyperinflation and suctioning to manage secretions and prevent ventilator-associated pneumonia. While the effects of manual chest vibration on mucus clearance are strongly controversial (66), positioning can help lung recruitment, promote weaning and, in this case, could have enhanced the efficacy of MH.

In recent years, there has been a growing interest in automatic systems aiming at promoting standardized weaning strategies (30, 67). Such automated systems included in ventilators software has been shown to significantly reduce the weaning time in critically ill patients (68). However, different authors showed that respiratory physiotherapy–driven weaning protocols can further decrease weaning time, probably due to a more efficient assessment and management of intercurrent situations and individual variability during the process. These results emphasized the need for personalized therapeutic interventions combined with continuous monitoring of the patient response to guarantee a rapid and precise support in the complex rehabilitation framework of MV patients (68). In this scenario, a specific patient’s stratification might be crucial to better standardize the optimal therapeutic strategies in acute patients admitted to ICU (41).

Our findings highlighted the lack of data about the long-term outcomes of post-weaned patients and the eventual long-term advantages of rehabilitative interventions are far from being fully understood. Therefore, it is mandatory to emphasize the role of physiotherapy in weaning optimization, reducing MV complications and immobilization consequences in terms of functional outcomes, residual disability and increased social and health care costs (3, 5–8).

Taken together, the findings of the present systematic review of RCTs and meta-analysis highlight the efficacy of pulmonary rehabilitation strategies including early mobilization, ACTs and respiratory muscle training. However, our data underline a gap of knowledge about the optimal components of tailored pulmonary physiotherapy interventions in MV patients admitted to ICU. Future research should focus on precise patient stratifications to better characterize the synergism between different rehabilitative interventions, focusing resources and improving outcomes of MV patients admitted to ICU.

We are aware that the present systematic review is not free from limitations. First, several therapeutic approaches have been assessed with significant implications in terms of specificity of the study results. Second, due to the heterogeneity of the study included, it was not possible to assess the efficacy of single rehabilitation modality. Therefore, the optimal rehabilitation program is still uncertain given the lack of quantitative data about each single intervention. On the other hand, it should be noted that in clinical setting the pulmonary rehabilitation programs were composed by the integration of different rehabilitation strategies including a early mobilization, positioning, ACTs/lung expansion, respiratory muscle training and automatic weaning systems. Moreover, the aim of the study was to assess the role of comprehensive physiotherapy intervention; therefore, to the best of our knowledge, the present work represents the first systematic review of RCTs assessing an integrated rehabilitation intervention in line with the current clinical practice performed in patients in ICU setting without pulmonary chronic conditions.

In conclusions, the results of this systematic review of RCTs with meta-analysis supported the efficacy of a comprehensive physiotherapy intervention in reducing MV duration in critical ill patients without chronic respiratory conditions. The development of specific rehabilitation strategies, that could represent a valid therapeutic approach to improve the weaning process and reduce MV duration, is an urgent need not only to prevent the onset of severe complications, but also to ensure sustainability in terms of health care costs reduction.

In this scenario, the present work provided promising results about the role of early mobilization, positioning, airway clearance techniques, lung expansion and respiratory muscle training in the complex framework of mechanically ventilated critical ill patients.

Further studies are needed to better characterize the effects of specific rehabilitation strategies to reduce MV duration and optimize the weaning process in order to improve the best rehabilitative intervention in critical ill patients without chronic respiratory conditions.

## Data Availability Statement

The raw data supporting the conclusions of this article will be made available by the authors, without undue reservation.

## Author Contributions

LL, AS, FD’A, and MI contributed to study design and conceptualization. LL and FD’A contributed to databases searching. LL, FD’A, and MI contributed to data screening and data extraction. LL, AS, and FD’A contributed to data synthesis, interpretation, and manuscript drafting. NM contributed to statistical analysis. CM and MI contributed to critical revision. BP, NM, LC, and AA contributed to visualization. AS, AA, CM, and MI contributed to study supervision. LL contributed to study submission. All authors read and approved the final version of the manuscript.

## Conflict of Interest

The authors declare that the research was conducted in the absence of any commercial or financial relationships that could be construed as a potential conflict of interest.

## Publisher’s Note

All claims expressed in this article are solely those of the authors and do not necessarily represent those of their affiliated organizations, or those of the publisher, the editors and the reviewers. Any product that may be evaluated in this article, or claim that may be made by its manufacturer, is not guaranteed or endorsed by the publisher.

## Footnotes

1
https://www.crd.york.ac.uk/prospero

2
https://www.crd.york.ac.uk/prospero/display_record.php?ID=CRD42022299537
