# Supplementary material for: Efficacy of Physiotherapy Interventions on Weaning in Mechanically Ventilated Critically Ill Patients: A Systematic Review and Meta-Analysis
Source: Front Med (Lausanne). 2022 May 9;9:889218. doi: 10.3389/fmed.2022.889218 (PMC9124783; doi:10.3389/fmed.2022.889218)
Supplement: Supplementary file 1 [file Table_1.DOCX]

| **Supplementary Table 1.** Search strategy. |
| --- |
| ***PubMed***  ("mechanical ventilation"[All Fields] OR "mechanical respiration"[All Fields] OR "Artificial ventilation"[All Fields] OR "Artificial respiration"[All Fields]) AND ("weaning "[MeSH Terms] OR "weaning"[All Fields] OR "Ventilator Weaning"[All Fields]) AND ("rehabilitation"[MeSH Terms] OR "rehabilitation"[All Fields] OR "physiotherapy"[All Fields] OR "mobilization"[All Fields] OR "early mobilization"[All Fields] OR "pronation"[All Fields] OR "posture"[All Fields] OR "airway clearance"[All Fields] OR "respiratory muscle training"[All Fields] OR "inspiratory muscle training"[All Fields] OR "strength training"[All Fields] OR "resistance training"[All Fields] OR "strength exercise"[All Fields] OR "resistance exercise"[All Fields] OR "training"[All Fields] OR "exercise"[MeSH Terms] OR "exercise"[All Fields] OR "physical exercise"[All Fields] OR "physical therapy"[All Fields]) |
| ***Scopus***  TITLE-ABS-KEY (((mechanical ventilation) OR (mechanical respiration) OR (Artificial ventilation) OR (Artificial respiration)) AND ((weaning) OR (Ventilator Weaning)) AND ((rehabilitation) OR (physiotherapy) OR (mobilization) OR (early mobilization) OR (pronation) OR (posture) OR (airway clearance) OR (respiratory muscle training) OR (inspiratory muscle training) OR (strength training) OR (resistance training) OR (strength exercise) OR (resistance exercise) OR (training) OR (exercise) OR (Physical exercise) OR (physical therapy))) |
| ***Web of Science***  TS=(((mechanical ventilation) OR (Artificial ventilation)) AND ((weaning) OR (Ventilator Weaning)) AND ((rehabilitation) OR (physiotherapy) OR (mobilization) OR (early mobilization) OR (pronation) OR (posture) OR (airway clearance) OR (respiratory muscle training) OR (inspiratory muscle training) OR (strength training) OR (resistance training) OR (strength exercise) OR (resistance exercise) OR (training) OR (exercise) OR (Physical exercise) OR (physical therapy))) |
| ***Cochrane Central Register of Controlled Trials (CENTRAL):***  #1 MeSH descriptor: [Mechanical Ventilation] explode all trees  #2 MeSH descriptor: [Artificial Ventilation] explode all trees  #3 MeSH descriptor: [Weaning] explode all trees  #4 MeSH descriptor: [Rehabilitation] explode all trees  #5 MeSH descriptor: [Mobilization] explode all trees  #6 MeSH descriptor: [Training] explode all trees  #7 MeSH descriptor: [Exercise] explode all trees  #8 (#1 OR #2) AND #3 AND (#4 OR #5 OR #6 OR #7) |
| ***Physiotherapy Evidence Database (PEDro):***  Mechanical Ventilation Weaning Rehabilitation |
