# Supplementary material for: Efficacy of Physiotherapy Interventions on Weaning in Mechanically Ventilated Critically Ill Patients: A Systematic Review and Meta-Analysis
Source: Front Med (Lausanne). 2022 May 9;9:889218. doi: 10.3389/fmed.2022.889218 (PMC9124783; doi:10.3389/fmed.2022.889218)
Supplement: Supplementary file 2 [file Table_2.DOCX]

| Supplementary Table 2. Characteristics of the studies assessed in full text and excluded. | |
| --- | --- |
| *Study* | ***Reason for exclusion*** |
| Aggarwal et al. 2009 | No rehabilitation treatment |
| Andreu et al. 2019 | No rehabilitation treatment |
| Bailey et al. 2009 | Not randomized controlled trial |
| Ballesteros Reviriego 2020 | Not randomized controlled trial |
| Bissett et al. 2016 | Patients assessed after weaning |
| Bissett et al. 2019 | Patients assessed after weaning |
| Black et al. 2012 | Not randomized controlled trial |
| Borup et al. 2019 | Not randomized controlled trial |
| Bösel et al. 2012 | Not randomized controlled trial |
| Carenco & Tran-Van 2014 | Not in English language |
| Caruso et al. 2005 | Patients with chronic conditions |
| Castle et al. 2011 | Not randomized controlled trial |
| Castro et al. 2013 | Not randomized controlled trial |
| Chen et al. 2012 | Patients with chronic conditions |
| Chen et al. 2018 | Not randomized controlled trial |
| Chen et al. 2019 | Patients with chronic conditions |
| Chittawatanarat & Thongchai 2009 | Not randomized controlled trial |
| Clini et al. 2006 | Patients with chronic conditions |
| Condessa et al. 2013 | Patients with chronic conditions |
| Crespi et al. 2009 | Not in English language |
| da Silva Guimaraes et al. 2021 | Patients with chronic conditions |
| Daniel et al. 2015 | Not randomized controlled trial |
| de Beer et al. 2018 | Not randomized controlled trial |
| Delgado et al. 2019 | Patients with chronic conditions |
| Dziewas et al. 2017 | Non-ventilated patients |
| Ebadi et al. 2015 | No rehabilitation treatment |
| Fenton et al. 2016 | No rehabilitation treatment |
| Forni et al. 2020 | Not randomized controlled trial |
| Fossat et al. 2021 | Not randomized controlled trial |
| Giraldo et al. 2006 | Not randomized controlled trial |
| Gnanapandithan et al. 2011 | Patients with chronic conditions |
| Gonçalves et al. 2012 | Patients with chronic conditions |
| Grasso et al. 2000 | Not randomized controlled trial |
| Guimarães et al. 2020 | Patients with chronic conditions |
| Gust et al. 1996 | Patients assessed after weaning |
| Gutierrez et al. 2010 | Patients with chronic conditions |
| Herdy et al. 2008 | Non-ventilated patients |
| Hermans et al. 2014 | Patients with chronic conditions |
| Hoff et al. 2014 | Patients with chronic conditions |
| Huang et al. 2014 | Patients with chronic conditions |
| Ibrahim and Mohamed 2018 | No weaning outcome |
| Jones et al. 2013 | Patients with chronic conditions |
| Kimmoun et al. 2015 | Not randomized controlled trial |
| Kirakli et al. 2015 | Patients with chronic conditions |
| Mancebo et al. 2006 | No weaning outcome |
| Müller et al. 2006 | No weaning outcome |
| Navalesi et al. 2008 | No rehabilitation treatment |
| Lago Borges et al. 2014 | Patients with chronic conditions |
| Lesage et al. 2016 | Not in English language |
| Liu et al. 2020 | Patients with chronic conditions |
| Mehani et al. 2020 | No peer-reviewed journal |
| Modawal et al. 2002 | Not randomized controlled trial |
| Pathak et al. 2021 | Not randomized controlled trial |
| Paulus et al. 2011 | No weaning outcome |
| Porta et al. 2005 | Patients with chronic conditions |
| Prasad et al. 2021 | Patients with chronic conditions |
| Rezaiguia-Delclaux et al. 2021 | No rehabilitation treatment |
| Roustan et al. 1992 | Patients with chronic conditions |
| Routsi et al. 2010 | Patients with chronic conditions |
| Scaramuzzo et al. 2021 | Not randomized controlled trial |
| Schonhofer 1997 | Not in English language |
| Schreiber et al. 2019 | Not randomized controlled trial |
| Shaikh et al. 2014 | Not randomized controlled trial |
| Shen et al. 2017 | Patients with chronic conditions |
| Shinoda et al. 2018 | Not randomized controlled trial |
| Sibilla et al. 2020 | Not randomized controlled trial |
| Skinner et al. 2009 | Not randomized controlled trial |
| Smith et al. 2014 | Not randomized controlled trial |
| Staudinger et al. 2010 | Patients with chronic conditions |
| Tanios et al. 2006 | No rehabilitation treatment |
| Templeton & Palazzo 2007 | Patients with chronic conditions |
| Thammata et al. 2021 | No weaning outcome |
| Thomas et al. 2014 | No weaning outcome |
| Twose et al. 2019 | Patients assessed after weaning |
| Vadi et al. 2021 | Not randomized controlled trial |
| Vagheggini et al. 2013 | Patients with chronic conditions |
| Vitacca et al. 2016 | Patients with chronic conditions |
| Wilkinson et al. 2021 | Not randomized controlled trial |
| Xiao et al. 2020 | Patients with chronic conditions |
| Zhang et al. 2020 | Not randomized controlled trial |
